# Integrative Mendelian Randomization and Single‐Cell Pseudotime Analysis Reveal DKK3 as a PI3K–AKT‐Modulated Driver of Esophageal Squamous Cell Carcinoma

**DOI:** 10.1155/humu/6777692

**Published:** 2026-03-24

**Authors:** Zhanghao Huang, Tiegang Cao, You Lang Zhou, Jiahai Shi

**Affiliations:** ^1^ Medical School of Nantong University, Nantong University, Nantong, Jiangsu, China, ntu.edu.cn; ^2^ Department of Thoracic Surgery, Affiliated Hospital of Nantong University, Nantong, Jiangsu, China, ahnmc.com; ^3^ Nantong Key Laboratory of Translational Medicine in Cardiothoracic Diseases, And Research Institution of Translational Medicine in Cardiothoracic Diseases, Affiliated Hospital of Nantong University, Nantong, Jiangsu, China, ahnmc.com; ^4^ Research Center of Clinical Medicine, Affiliated Hospital of Nantong University, Nantong, Jiangsu, China, ahnmc.com

**Keywords:** DKK3, esophageal squamous cell carcinoma, Mendelian randomization, PI3K–AKT signaling pathway, single-cell analysis

## Abstract

**Background:**

Esophageal squamous cell carcinoma (ESCC) remains a highly lethal malignancy, and the molecular drivers of its progression are not fully defined. Dickkopf‐3 (DKK3), a context‐dependent modulator of oncogenic signaling, has been implicated in several solid tumors, but its role in ESCC is unclear.

**Methods:**

We integrated Mendelian randomization based on cis‐expression quantitative trait loci (cis‐eQTLs) from 31,684 individuals with The Cancer Genome Atlas (TCGA) ESCC transcriptomic data (|log^2^FC| > 1 and *p* < 0.05) , single‐cell RNA sequencing, and in vitro/in vivo functional assays to identify causal ESCC‐associated genes. DKK3 expression was validated by Western blotting, its effects on ESCC cell proliferation, migration, and clonogenicity were tested experimentally, and DKK3‐related pathways were explored by Gene Ontology (GO) and Kyoto Encyclopedia of Genes and Genomes (KEGG) analyses.

**Results:**

Integrative differential expression and MR analyses identified 22 ESCC‐associated genes. Single‐cell epithelial profiling further prioritized three candidates (DKK3, NINJ2, and SAPCD2), among which only DKK3 showed high and progressively increasing expression along the malignant epithelial trajectory and was therefore selected for mechanistic validation. DKK3 knockdown significantly inhibited ESCC cell proliferation, migration, and clonogenic capacity in vitro and suppressed tumor growth in nude mice. DKK3 silencing reduced p‐PI3K and p‐AKT levels, and enrichment analyses supported that DKK3 promotes ESCC progression at least partly through activation of the PI3K–AKT signaling pathway.

**Conclusion:**

By linking germline regulatory variation with single‐cell tumor profiling and functional validation, this study identifies DKK3 as a causally relevant oncogenic regulator in ESCC that drives epithelial tumor growth and migration via PI3K–AKT pathway activation, supporting DKK3 as a potential biomarker and therapeutic targe

## 1. Introduction

ESCC is a highly lethal malignancy with marked geographic variation in incidence and limited therapeutic options [1]. Despite continuous advances in surgery, radiotherapy, and chemotherapy, the prognosis of many patients remains unsatisfactory, highlighting the urgent need to elucidate the molecular drivers underlying ESCC initiation and progression. Aberrant gene expression plays a pivotal role in tumor development. For example, overexpression of human epidermal growth factor receptor 2 (HER2) in breast cancer and epidermal growth factor receptor (EGFR) in nonsmall cell lung cancer is closely associated with increased tumor aggressiveness and poor prognosis [2, 3]. Similarly, activation of BRAF in melanoma and dysregulation of MYCN and CCND1 in colorectal cancer significantly promote tumor proliferation and metastasis [4, 5]. These examples illustrate how abnormal gene expression profiles can disrupt normal regulation of cell proliferation and apoptosis, thereby driving malignant transformation.

Dickkopf WNT signaling pathway inhibitor 3 (DKK3) encodes a widely expressed secreted glycoprotein that modulates Wnt and other oncogenic signaling pathways. In ESCC, oral cancer and colorectal cancer, DKK3 overexpression has been reported to enhance tumor cell proliferation and migration [6–9]. However, the biological role of DKK3 is clearly context‐dependent: in different tumor types and microenvironments, DKK3 can act either as a tumor promoter or as a tumor suppressor [10–12]. In ESCC, most existing studies are based on bulk transcriptomic data and conventional functional assays, and the causal relationship between DKK3 and ESCC risk, its cell–type‐specific expression patterns, and its dynamic changes during tumor evolution remain incompletely understood.

To overcome the limitations of traditional association studies, we adopted an integrative strategy that combines genetic epidemiology with high‐resolution transcriptomics. Using cis‐expression quantitative trait locus (cis‐eQTL)–based Mendelian randomization (MR), we inferred the causal relationship between gene expression and ESCC risk from the perspective of germline genetic regulation [13, 14]. In parallel, we integrated bulk RNA sequencing with single‐cell RNA sequencing (scRNA‐seq) data to dissect cellular heterogeneity, identify key cellular subpopulations, and reconstruct differentiation trajectories within ESCC at single‐cell resolution [15, 16]. By linking cis‐eQTL–MR findings with bulk and single‐cell transcriptomic information, we established a framework that connects germline regulatory variation to specific cell types and dynamic cellular states in ESCC, enabling prioritization of genes with putative causal roles in this malignancy.

**Figure 1:**
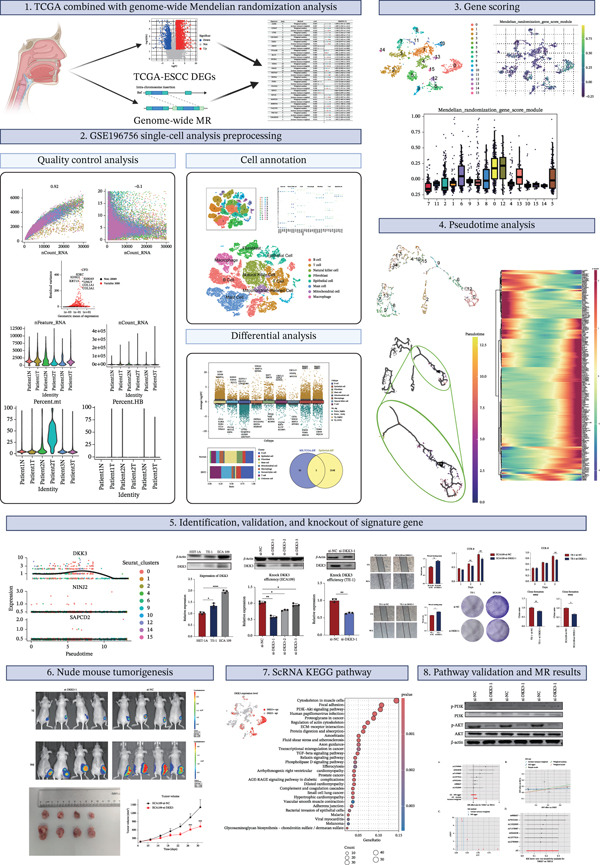
Study flowchart. Overall analytical and experimental workflow of the study, from data acquisition and bioinformatic screening to single‐cell analysis and functional validation.

To date, studies of DKK3 in ESCC have largely been confined to bulk‐level analyses, and no work has combined MR with scRNA‐seq and pseudotime trajectory analysis to systematically evaluate the causal contribution and temporal dynamics of DKK3 in ESCC. Building on this background, we integrated cis‐eQTL–based MR, bulk differential expression analysis, scRNA‐seq and pseudotime trajectory analysis, together with in vitro and in vivo functional experiments, to define the causal role of DKK3 in ESCC development and to elucidate its downstream molecular mechanisms, thereby providing new evidence for ESCC biomarker discovery and potential targeted therapeutic strategies (Figure 1).

## 2. Materials and Methods

### 2.1. Data Sources

We employed a multidimensional approach to investigate the relationship between genes and ESCC. We used cis‐eQTL summary statistics from the eQTLGen consortium and, by combining them with ESCC genome‐wide association study (GWAS) data in MR analyses, identified genes whose genetically predicted expression is associated with ESCC risk. This allowed us to preliminarily screen out genes potentially related to the disease. To achieve a comprehensive understanding, we utilized cancer genome data from The Cancer Genome Atlas (TCGA) project and scRNA‐seq data from the Gene Expression Omnibus (GEO) database for detailed analysis. We identified genes with significant expression changes in ESCC. By combining genetic screening with large‐scale genomic and single‐cell data, we comprehensively studied ESCC from genetic to molecular levels.

### 2.2. Animals

Eight specific pathogen‐free (SPF) male BALB/c‐nu mice (42–56 days old) were obtained from Nantong University (Nantong, China). All experimental procedures involving animals were approved by the Institutional Animal Care and Use Committee of Nantong University (IACUC No. AUP‐QY‐C‐S‐2024‐022) and conducted in accordance with institutional and national guidelines for the care and use of laboratory animals.

### 2.3. Protein Extraction and Western Blotting

Cells or tissues were lysed on ice in RIPA buffer supplemented with PMSF (Thermo Fisher Scientific, Shanghai, China). After centrifugation at 12,000 × g for 15 min at 4°C, supernatants were collected and protein concentrations were determined by BCA assay. Equal amounts of protein were mixed with 5 × sodium dodecyl sulfate–polyacrylamide gel electrophoresis (SDS‐PAGE) loading buffer (Servicebio, Wuhan, China), boiled at 95°C for 5 min, separated by SDS‐PAGE using a dual‐color prestained protein marker (Epizyme, Beijing, China), and transferred onto polyvinylidene fluoride (PVDF) membranes (Sigma‐Aldrich, United States). Membranes were blocked with protein‐free rapid blocking solution (Servicebio, Wuhan, China), incubated with primary antibodies at 4°C overnight and HRP‐conjugated secondary antibodies at room temperature for 1 h. Protein bands were visualized by enhanced chemiluminescence.

### 2.4. Identification of Differentially Expressed Genes

We utilized the TCGA database to identify differentially expressed genes, providing clues for the study of cancer molecular mechanisms and the discovery of potential biomarkers. By conducting an in‐depth analysis of the transcriptome data from cancer and control samples in the TCGA database, we applied strict screening criteria. We required the absolute value of log2 fold change (log2FC) to be greater than 1 (log2FC > 1 or < −1) and the *p* value to be less than 0.05. This ensured that the identified differentially expressed genes had significant statistical and biological relevance. Ultimately, we successfully identified 7133 differentially expressed genes for further experiments.

### 2.5. Genome‐Wide MR

We then leveraged the eQTLGen consortium, which provided extensive data resources from a comprehensive meta‐analysis of expression quantitative trait loci (eQTLs) in peripheral blood samples from 31,684 individuals. Using cis‐eQTLs with significant statistical significance, we constructed a genetic proxy model covering 3295 differentially expressed genes. We further focused on 2619 eQTLs with key regulatory roles within a 100‐kb genomic window, laying the foundation for subsequent in‐depth gene function exploration and disease association analysis.

Subsequently, we employed the twoSampleMR R package to conduct MR analysis using GWAS summary statistics from a large database containing 476,306 European individuals (ebi‐a‐GCST90018841). During the analysis, we excluded single nucleotide polymorphisms (SNPs) with insufficient statistical power and assessed the scientific applicability and accuracy of the selected instrumental variables based on the 1000 Genomes European reference panel, providing a reliable basis for subsequent causal inference.

To assess causal direction and minimize reverse causation (ESCC affecting gene expression), we applied Steiger filtering to retain instruments consistent with gene expression influencing ESCC risk. In the MR estimation step, we first used the classic Wald ratio method for preliminary estimation and then integrated multiple meta‐analysis methods, including inverse variance weighted (IVW), MR‐Egger, and weighted median models, to comprehensively analyze the data. We used Bonferroni correction to complete the sensitivity analysis and multiple testing steps, setting the significance threshold at *p* < 10^−8^. This high standard ensured that the genetic tools identified were highly significant statistically, thereby ensuring the strong reliability and applicability of the conclusions drawn in this study.

### 2.6. Single‐Cell Analysis

We acquired scRNA transcriptomic data from the GEO, specifically focusing on from GSM5900215 to GSM5900220 (GSE196756). To ensure data quality, we used the Seurat toolkit to process the raw data. During quality control, we applied strict criteria to filter the data. For example, to remove erythrocyte contamination, we identified and removed genes related to hemoglobin and calculated their expression percentage as an important quality control indicator.

In the data processing stage, we analyzed the relationships between these indicators and then normalized the data using the “LogNormalize” method. We selected the Top 2000 most variable genes and further processed the data using “ScaleData” and “SCTransform” methods to reduce technical errors. We performed PCA to reduce the data to 50 principal components and used an elbow plot to determine that the first 29 principal components were suitable for analysis. Using the FindNeighbors and FindClusters functions, we clustered the cells into five groups based on the first 29 principal components. We then used the Uniform Manifold Approximation and Projection (UMAP) algorithm for dimensionality reduction and visualization. We identified differentially expressed genes with |log2FC| > 1 and *p* value <0.05, providing clues for studying the biological function differences between cell clusters. Module scores were calculated using the AddModuleScore function implemented in the Seurat package with default parameters. For each cell, we defined gene sets representing the epithelial program and other relevant functional modules. AddModuleScore computes, for each gene set, the averaged expression of the genes in the set after subtracting the aggregated expression of control gene sets matched for expression level, yielding a relative module score per cell. Higher scores therefore indicate stronger activation of the corresponding gene program. For downstream analyses, we used these module scores to compare epithelial features across cell clusters and conditions.

### 2.7. Gene Scoring Analysis

First, we read the three characteristic genes that we had identified. We then used the AddModuleScore function in the Seurat package to calculate the module scores for these genes and stored the scores in a designated slot of the Seurat object. Subsequently, we modified the metadata column name of the Seurat object to assign an easily recognizable name to the module scores. Finally, we used the DimPlot function to create a UMAP dimensionality reduction plot, with data points colored according to module scores to visually display the distribution of different cell populations.

### 2.8. Single‐Cell Pseudotime Analysis

We primarily utilized the Seurat and Monocle3 R packages. First, we extracted gene expression data (counts layer) from the Seurat object and created a new cell_data_set object, which included cell metadata and gene metadata. Next, we performed data preprocessing, including normalization and dimensionality reduction, and assessed data variability using a PCA variance explanation plot. We then applied UMAP for nonlinear dimensionality reduction and plotted the resulting cell distribution. For clustering analysis, we used the Louvain algorithm and visualized the clustering results. Additionally, we inferred cell developmental trajectories by learning the cell network graph. To identify genes associated with the trajectory, we used graph‐testing methods to recognize significant genes and selected the Top 200 for further analysis. We also smoothed and normalized the gene expression data and performed Ward.D2 hierarchical clustering to identify similarities in gene expression patterns. Finally, we selected significant genes and used functions from the Monocle3 package to plot their expression trends in pseudotime and the distribution of cells along the trajectory, revealing dynamic changes during cell development.

### 2.9. Functional Enrichment Analysis of Characteristic Genes

After identifying the characteristic genes, we conducted in‐depth differential analysis based on their expression in epithelial cells. We selected genes with significant differences. To comprehensively evaluate the potential involvement and mechanisms of these DEGs in various biological processes (BP) and signaling pathways, we utilized R packages such as clusterProfiler, enrichplot, and DOSE for functional enrichment analysis. These analyses included GO and KEGG pathways to find the biological roles of the DEGs.

### 2.10. Preparation of si‐DKK3

Based on the human DKK3 gene sequence, three types of double‐stranded DKK3‐siRNA were designed. We first conducted in vitro cell transfection experiments to identify the most effective siRNA in suppressing DKK3 expression among the three. Each siRNA consists of two strands and includes a nonspecific sequence as a control (negative siRNA). These siRNAs were commercially synthesized by Shanghai GenePharma Co., Ltd. After annealing treatment, they were converted into stable double‐stranded oligonucleotides. The measured molecular weight deviated from the theoretical value by no more than 0.05%, ensuring their precision.

### 2.11. Cell Viability Assay Using Cell Counting Kit‐8 (CCK‐8)

The TE‐1 and ECA109 cell lines, alongside their corresponding DKK3‐depleted derivatives, were inoculated into 6‐well plates at a density of 30000 cells per well. At 48, 72, and 96 h postseeding, 10 *μ*L of a CCK‐8 assay reagent was introduced to each well, then the plates were incubated at 37°C for an additional 2 h. Cell growth was quantified by measuring the absorbance at 450 nm using a spectrophotometer.

### 2.12. Wound Healing Assay

TE‐1 and ECA109 cells (control) and their DKK3‐knockout variants (experimental) were inoculated into 6‐well plates at 1 × 10^5^ cells per well. Upon achieving 80%–90% cell coverage, a scratch was made with a 1000 *μ*L pipette tip. Cells were cultured in serum‐free medium for 24 h. Scratch images were taken at 0 and 96 h to measure migration using the formula: (original scratch width—current scratch width)/original scratch width. This assessed the impact of DKK3 knockout on cell migration.

### 2.13. Clonogenic Assays

TE‐1 and ECA109 cells (control) and DKK3‐knockout variants (experimental) were seeded in 6‐well plates at 500 cells per well. Cells were cultured in complete medium for 14 days. After PBS washing to remove nonadherent cells, cells were treated with a 4% formalin solution for 15 min and then dyed using a 0.1% gentian violet solution for 30 min. Any surplus dye was washed away using purified water. Colonies were counted under a microscope to assess DKK3 knockout impact. Clonogenic assays rate = ((number of colonies)/(number of seeded cells)) × 100%.

### 2.14. Tumor Xenograft Experiment in Nude Mice

ECA109 cells and DKK3‐knockout ECA109 cells were injected subcutaneously into nude mice, and the mice were observed and recorded for 30 days. After cell injection, the health status and tumor growth of the mice were monitored, and changes in tumor size and shape were accurately recorded. Tumor volume was calculated using the formula: Tumor volume (mm^3 ) = ((length × width^2))/2.

Additionally, a real‐time imaging system was used to dynamically observe tumor growth and accurately define the growth curve. At the end of the experiment, the mice were euthanized, and the tumors were excised and measured for size.

### 2.15. Statistical Methods

A variety of statistical methods were comprehensively employed to ensure the scientific validity and reliability of the experimental results. The identification of differentially expressed genes in the TCGA database was based on the set criteria of log2FC and *p* value. The use of data from the eQTLGen consortium and MR analysis was combined with Bonferroni correction to identify genetic tools with significant significance. In single‐cell data analysis, the Seurat toolkit was used for preprocessing and quality control, and principal component analysis (PCA) and UMAP algorithms were applied for dimensionality reduction and clustering analysis to identify clusters with significantly differentially expressed genes. In cell experiments, unpaired *t*‐tests were used to analyze differences between treatment and control groups in CCK‐8 assays, wound healing assays, and clonogenic assays. In the tumor xenograft experiment in nude mice, tumor volume and weight were measured to assess tumor growth, and unpaired *t*‐tests were used to analyze differences between treatment and control groups.

## 3. Result

### 3.1. Differential Expression Analysis and MR

In this study, we collected seven normal tissue samples and 184 ESCC tumor tissue samples from TCGA database and performed differential gene expression analysis on these samples. Compared with normal samples, 1186 significantly downregulated genes and 5947 significantly upregulated genes were identified in cancer samples (Figure 2a). Through MR analysis, we screened out 231 genes that have a direct association with ESCC. By intersecting these two sets of genes, the results showed that three genes were significantly downregulated in cancer samples and had a negative direct correlation with ESCC (Figure 2b, 2c), whereas 19 genes were significantly upregulated and had a positive direct correlation with ESCC.

Figure 2Identification of ESCC‐associated genes by integrating differential expression and Mendelian randomization analyses. (a) Volcano plot showing differentially expressed genes (DEGs) between ESCC tumors and adjacent normal tissues. (b) Venn diagrams illustrating the overlap between DEGs and genes implicated by Mendelian randomization (MR). The left panel shows downregulated genes: The intersection between genes significantly downregulated in ESCC and genes with genetically predicted expression negatively associated with ESCC risk (three genes). The right panel shows upregulated genes: The intersection between genes significantly upregulated in ESCC and genes with genetically predicted expression positively associated with ESCC risk (19 genes). (c) Forest plot summarizing MR estimates for the 22 candidate genes.

**Figure:**
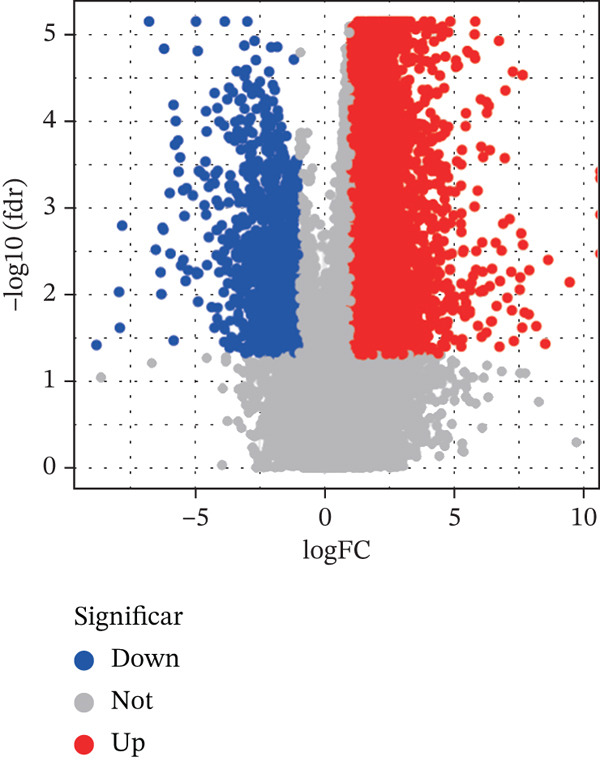
(a)

**Figure:**
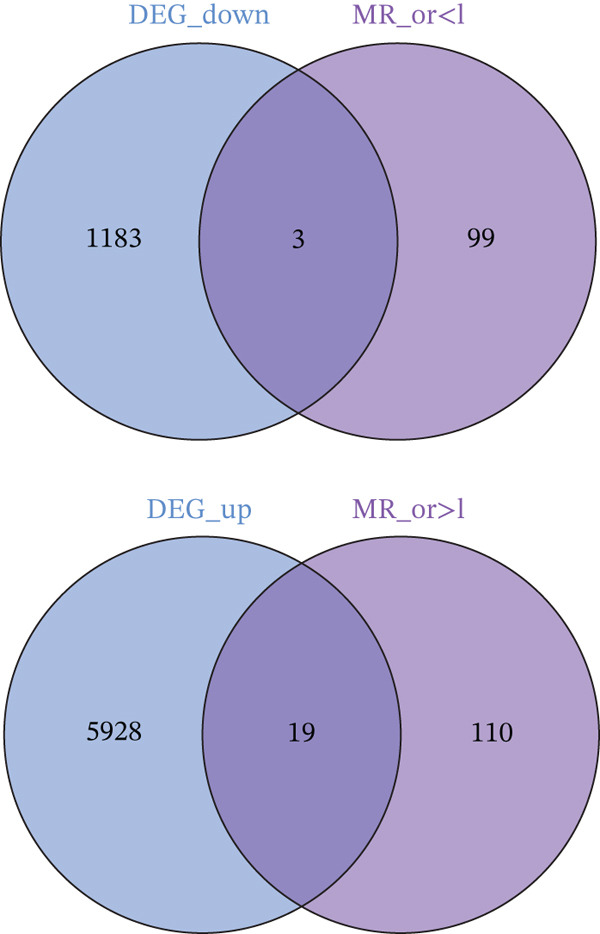
(b)

**Figure:**
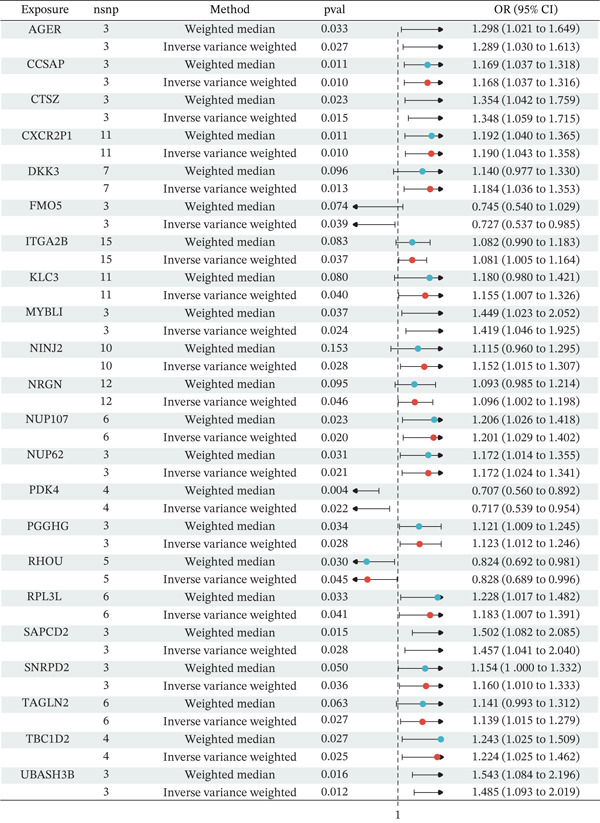
(c)

### 3.2. Quality Control and Dimensionality Reduction in Single‐Cell Analysis

A quality‐control scatter plot is used to examine total RNA and mitochondrial RNA proportions in individual cells, enabling the exclusion of low‐quality cells (Figure S1a). Figure S1b shows the relationship between the number of feature RNAs and the total RNA count in each cell, which helps to identify technical noise and biological variation among cells. Figure S1c is a plot of gene expression variability distribution, used to identify the most variable genes in the sample, which may be associated with biological differences. Figure S1d displays the distribution of gene expression across different samples, including the number of feature RNAs, total RNA count, mitochondrial RNA proportion, and proportion of highly expressed housekeeping genes. These analyses collectively provide a comprehensive overview of data quality and biological differences among cells, thereby laying the foundation for subsequent data analysis and biological interpretation.

To elucidate the cellular distribution and gene expression heterogeneity across patient samples, we applied PCA. The PCA plot visualizes cell distribution, with the *x*‐axis corresponding to the first principal component (PC1) and the *y*‐axis to the fortieth principal component (PC40). Distinct colors denote individual patients (Figure S1e). Additionally, we depicted the contribution of each principal component to data variation, with the *x*‐axis indicating principal component number and the *y*‐axis showing the corresponding standard deviation. This analysis highlights that the initial principal components capture the majority of data variation (Figure S1f).

Subsequent UMAP clustering revealed cell clustering results, with every point representing a single cell and different colors indicating distinct cell populations (Figure 3a). We also illustrated the UMAP space distribution of ESCC cells and normal cells. ESCC cells are marked in blue, whereas normal cells are in red, vividly contrasting their gene expression patterns (Figure 3b). Collectively, these visualizations offer crucial insights into cellular heterogeneity, population distribution characteristics, and cellular alterations under disease conditions.

Figure 3Single‐cell clustering and cell‐type annotation in ESCC and normal esophageal tissues. (a) UMAP projection of all single cells, with each point representing one cell and colors indicating unsupervised clusters. (b) UMAP visualization colored by sample group, highlighting the distribution of ESCC cells (blue) versus normal esophageal cells (red) in the shared embedding space, allowing direct comparison of their spatial distributions. (c) Dot plot of canonical marker genes across the annotated cell types. Dot size reflects the proportion of expressing cells, and color intensity reflects average expression. (d) UMAP plot showing the spatial distribution of the major cell types. Distinct colors denote different cell populations, including B cells (red), T cells (orange), NK cells (yellow), fibroblasts (green), epithelial cells (light green), mast cells (blue), mitochondria‐associated cells (purple), and macrophages (pink).

**Figure:**
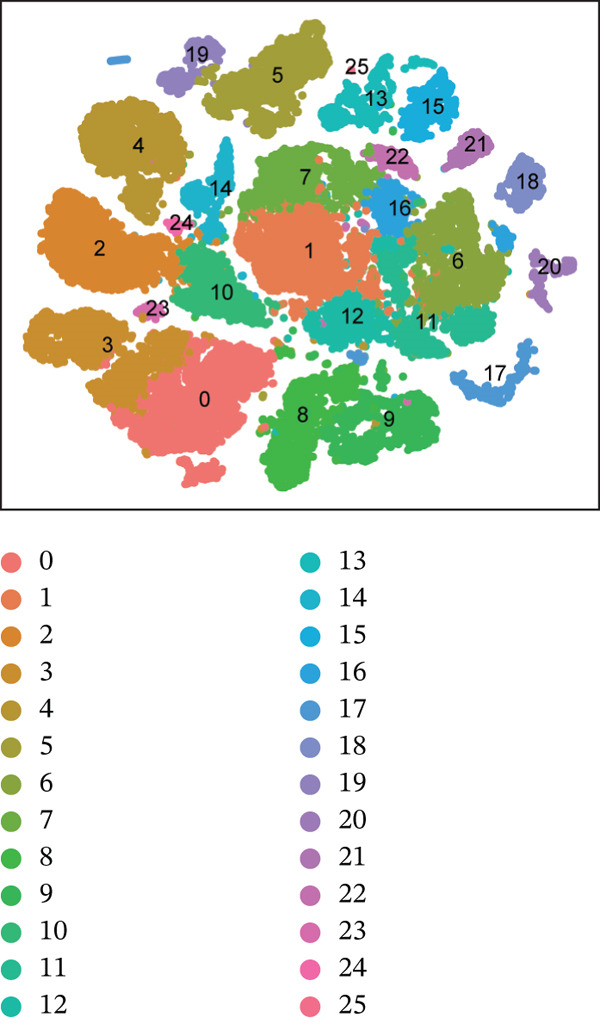
(a)

**Figure:**
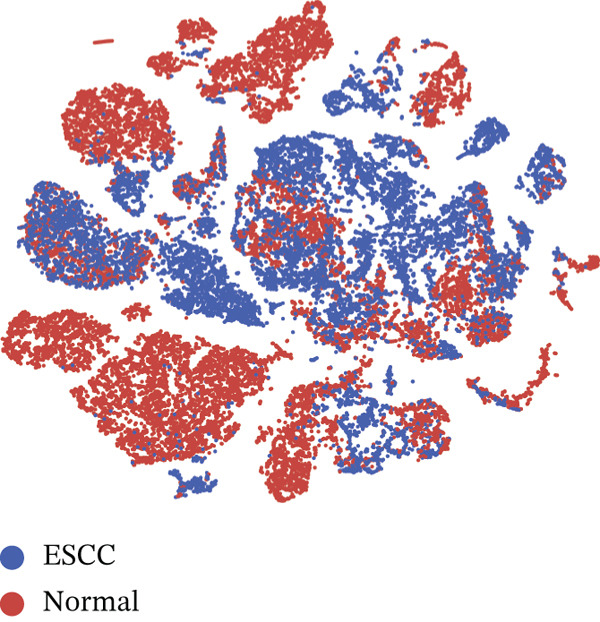
(b)

**Figure:**
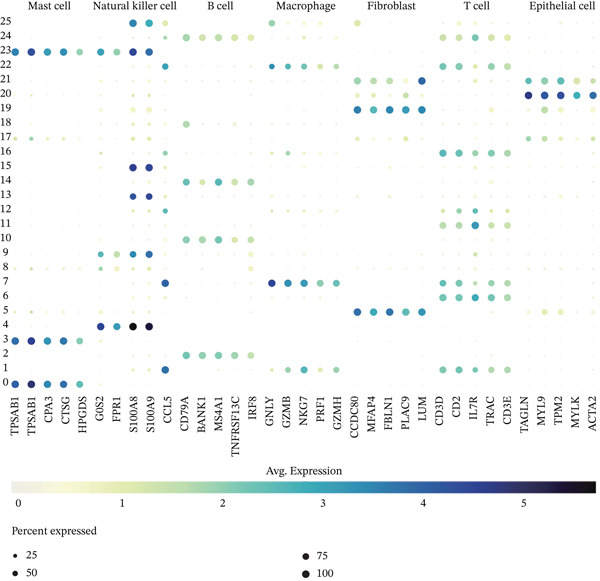
(c)

**Figure:**
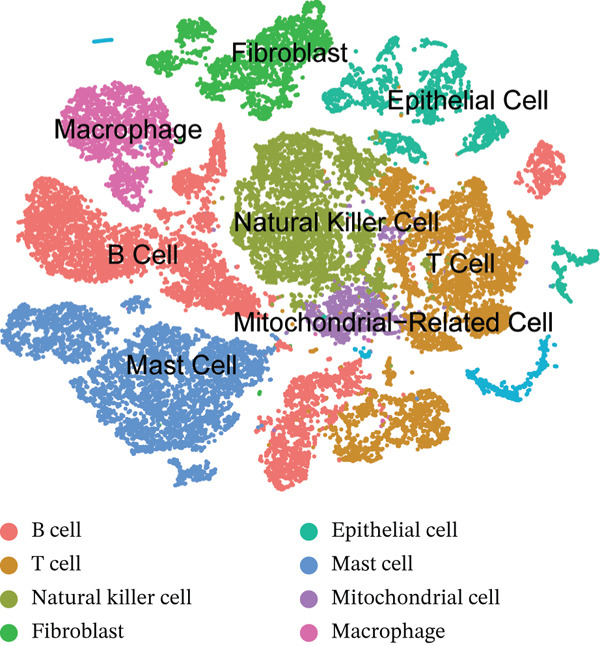
(d)

We initially assessed the average expression levels of specific genes across diverse cell types. Dot plots intuitively display gene expression differences among various cell types, including mast cells, natural killer cells, B cells, macrophages, fibroblasts, T cells, and epithelial cells. The dot size reflects the average gene expression level, whereas color intensity indicates the percentage of gene expression. This visualization method enables the clear identification of characteristic genes in each cell type, such as TSLP, GPR3, and CTN6 in mast cells; GZMA, GZMK, and FCGR3A in natural killer cells; and CD19, CD79A, and CD79B in B cells. This gene expression pattern analysis is highly significant for understanding the functions and characteristics of different cell types (Figure 3c).

Subsequently, we mapped the distribution of these cell types within the samples. Clustering analysis generated a distribution map, with different colored regions representing distinct cell types, including B cells, T cells, natural killer cells, fibroblasts, epithelial cells, mast cells, mitochondria‐related cells, and macrophages. This map facilitates the understanding of the relative proportions and spatial distribution of cell types within the samples, providing foundational data for further biological research (Figure 3d).

### 3.3. MR Gene Scoring and Visualization

By analyzing the gene expression patterns of between ESCC and normal tissue samples, we identified specific genes that were significantly upregulated or downregulated in these cell types (Figure 4a). We also observed marked discrepancies in the distribution of cellular categories between ESCC and normal tissue cells., indicating that certain cell types may undergo substantial changes in disease states (Figure 4b). By comparing the DEGs in epithelial cells between normal tissue and cancer tissue with the known genes in Figure 2d we identified three new genes potentially associated with ESCC: DKK3, NINJ2, and SAPCD2 (Figure 4c).

Figure 4Single‐cell expression landscape and MR scoring highlight cell‐type‐specific molecular features in ESCC. (a) Heatmap showing average expression of selected genes across major cell types. The *x*‐axis denotes cell types, the *y*‐axis denotes genes, and color indicates scaled expression levels. (b) Bar plot showing the proportional composition of major cell types in ESCC versus normal tissue samples, allowing comparison of cell‐type abundance under disease and nondisease conditions. (c) Venn diagram summarizing the overlap between ESCC DEGs and tumor‐related genes identified from TCGA and MR analyses. The plot displays ESCC‐specific DEGs (2151) and their overlaps with tumor‐related gene sets (16 and 3 genes, respectively). (d) UMAP embedding colored by unsupervised clusters (0–15), illustrating the spatial organization of transcriptionally distinct cell clusters in the reduced‐dimensional space. (e) UMAP embedding colored by MR score at the single‐cell level. Each dot represents a cell, with color intensity ranging from purple (low score) to yellow (high score), highlighting clusters with elevated MR scores. (f) Boxplots of MR scores across clusters. Each box represents one cell cluster, showing the median, interquartile range, and outliers. Comparison across clusters reveals which cell populations exhibit higher MR scores.

**Figure:**
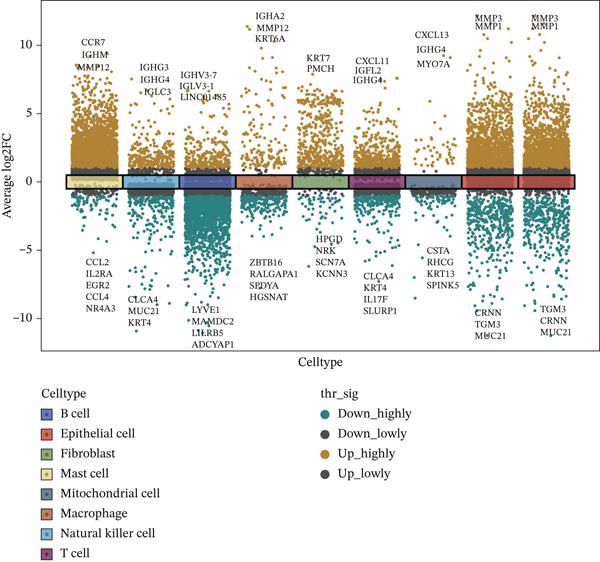
(a)

**Figure:**
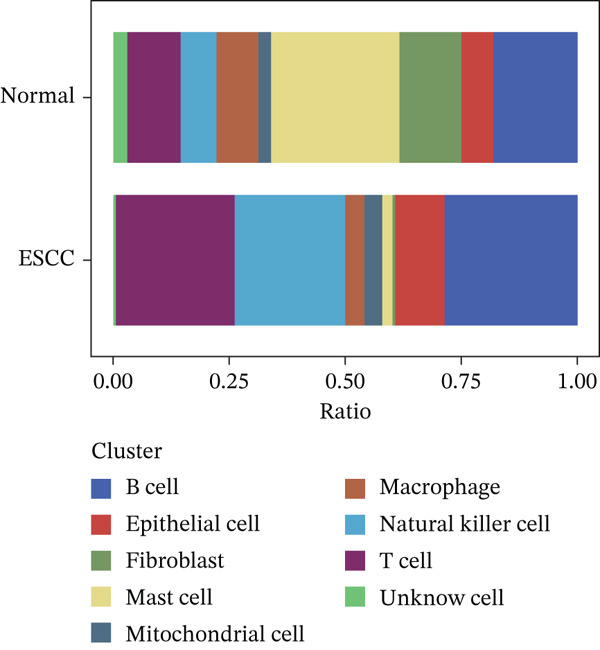
(b)

**Figure:**
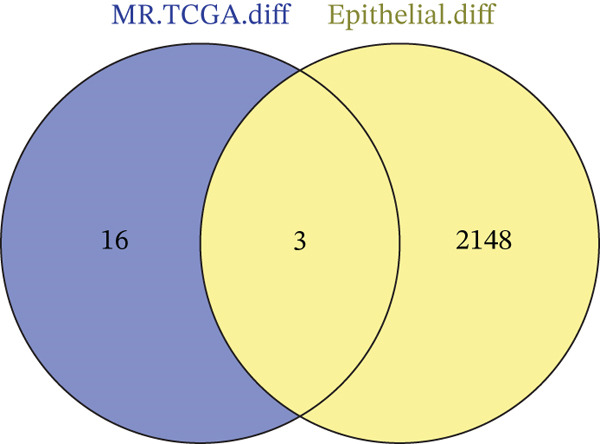
(c)

**Figure:**
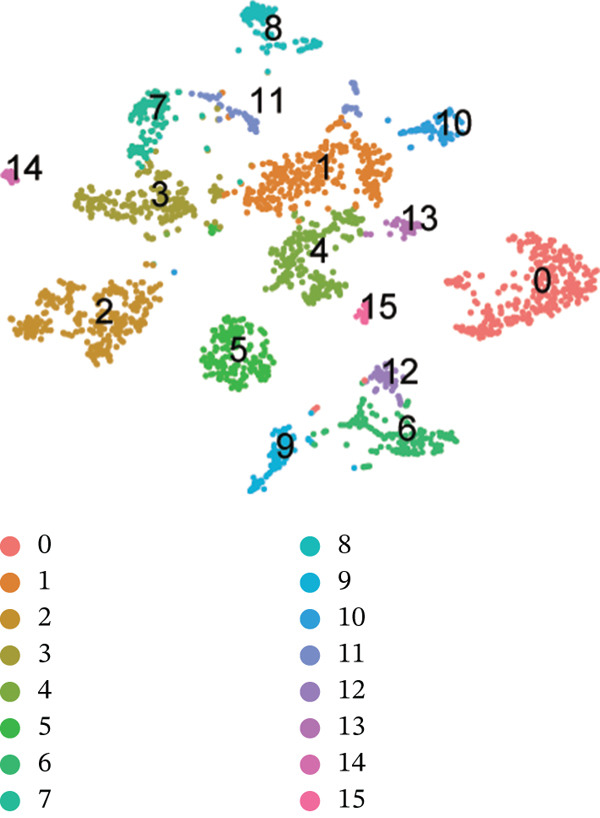
(d)

**Figure:**
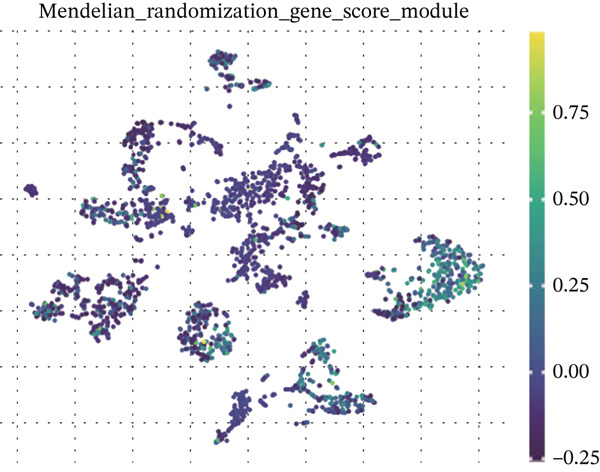
(e)

**Figure:**
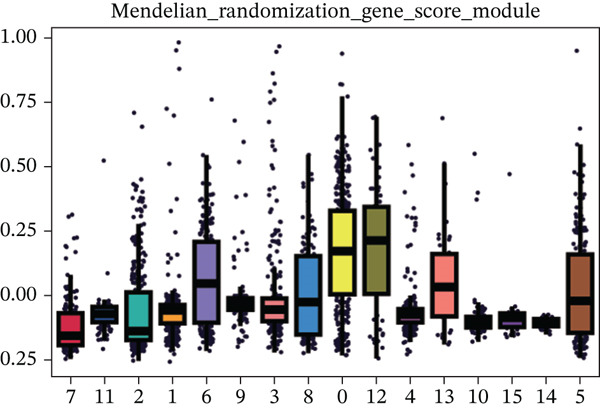
(f)

We conducted a detailed analysis of the gene expression profiles of epithelial cells in ESCC patients and identified three significantly highly expressed gene markers: DKK3, NINJ2, and SAPCD2. We used these three genes as MR scores to localize epithelial cells. First, we separately screened and performed dimensionality reduction on epithelial cells (Figure 4d) and generated a t‐SNE‐based dimensionality reduction map to visually observe the expression patterns of the gene scores (Figure 4e). Subsequently, we used bar charts to quantitatively analyze the gene expression levels in different cell clusters to identify high‐expression clusters (Figure 4f). Ultimately, we determined that Clusters 0 and 12 were the cell clusters with high MR scores. The identification of these high‐scoring cell clusters is of great value for understanding the molecular mechanisms of ESCC and exploring potential therapeutic targets.

### 3.4. Pseudotime Analysis and Identification of Feature Genes

We then performed pseudotime analysis on conventional epithelial cells and MR high‐scoring cell clusters (Clusters 0 and 12) to assess their different behaviors during the differentiation process. First, we demonstrated the inference of cell developmental trajectories, with different colors and numbers representing different cell clusters (0–15) to help us understand the transitions and relationships among cells during development (Figure 5a). Subsequently, we used color coding to represent “pseudotime” and displayed the distribution of cells in specific states. Green contours identified particular cell clusters or states, which are in similar stages of development. We set the starting point of time at the junction between MR high‐scoring cell clusters and other cells to better understand our needs (Figure 5b). We then focused on the expression levels of three genes (DKK3, NINJ2, and SAPCD2) in different cell clusters to see if they changed with cell differentiation (Figure 5c). The results showed that only DKK3 changed with differentiation, and thus we used the DKK3 gene for subsequent analysis. Finally, we visualized the expression of the Top 200 feature genes in each epithelial cell. The color indicates the *z*‐score, which is the normalized expression level. Each row represents a gene, and each column represents a cell. The color change (from blue to red) indicates the level of gene expression, forming a dynamic curve of gene expression changes to verify the accuracy of our pseudotime analysis of epithelial cells (Figure 5d).

Figure 5Developmental trajectories and gene expression dynamics revealed by single‐cell pseudotime analysis in ESCC. (a) UMAP plot with inferred developmental trajectories overlaid. Colors and numeric labels (0–15) denote distinct cell clusters. (b) UMAP plot colored by pseudotime, where the color gradient from purple to yellow represents progression along the inferred developmental trajectory. Green contours mark clusters or regions of cells in similar developmental states. (c) Pseudotime expression trends of DKK3, NINJ2, and SAPCD2 across different cell clusters. The *x*‐axis denotes pseudotime, and the *y*‐axis denotes normalized expression. Colored lines illustrate how the expression of these genes changes across developmental stages and clusters. (d) Heatmap of pseudotime‐associated genes. Each row corresponds to a gene and each column to a cell ordered by pseudotime. Color transitions from blue to red indicate low to high expression, visualizing coordinated gene expression programs along the trajectory.

**Figure:**
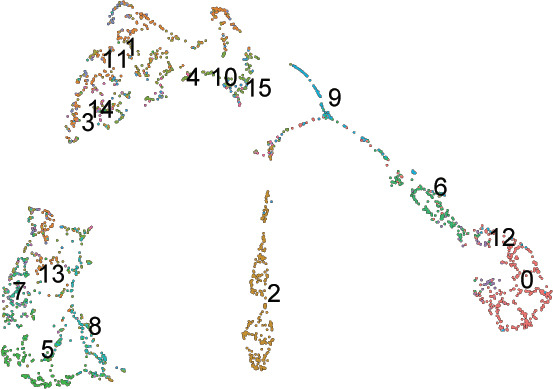
(a)

**Figure:**
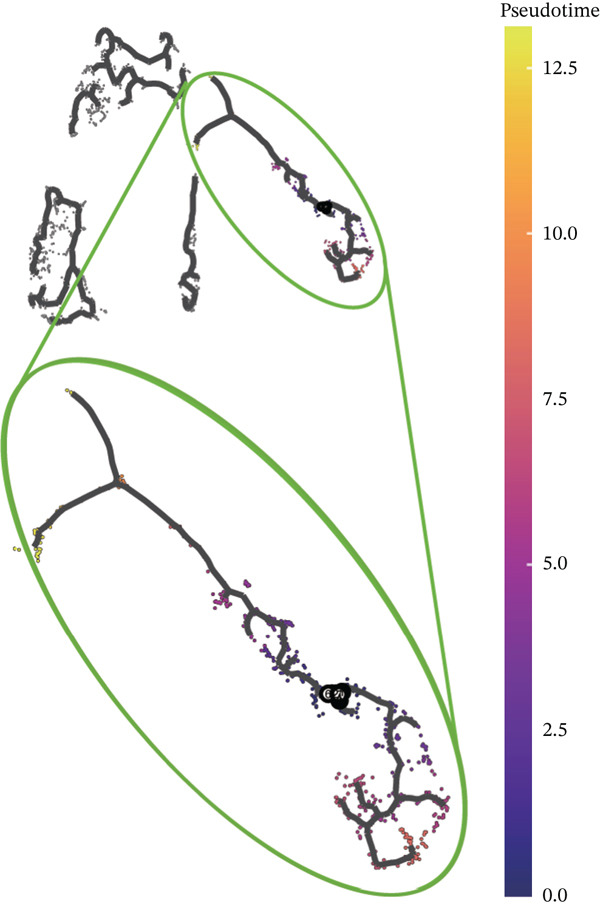
(b)

**Figure:**
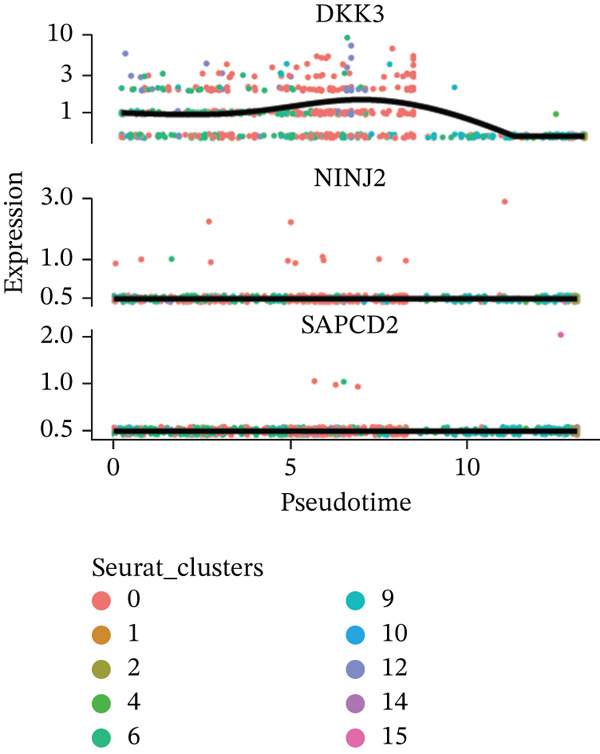
(c)

**Figure:**
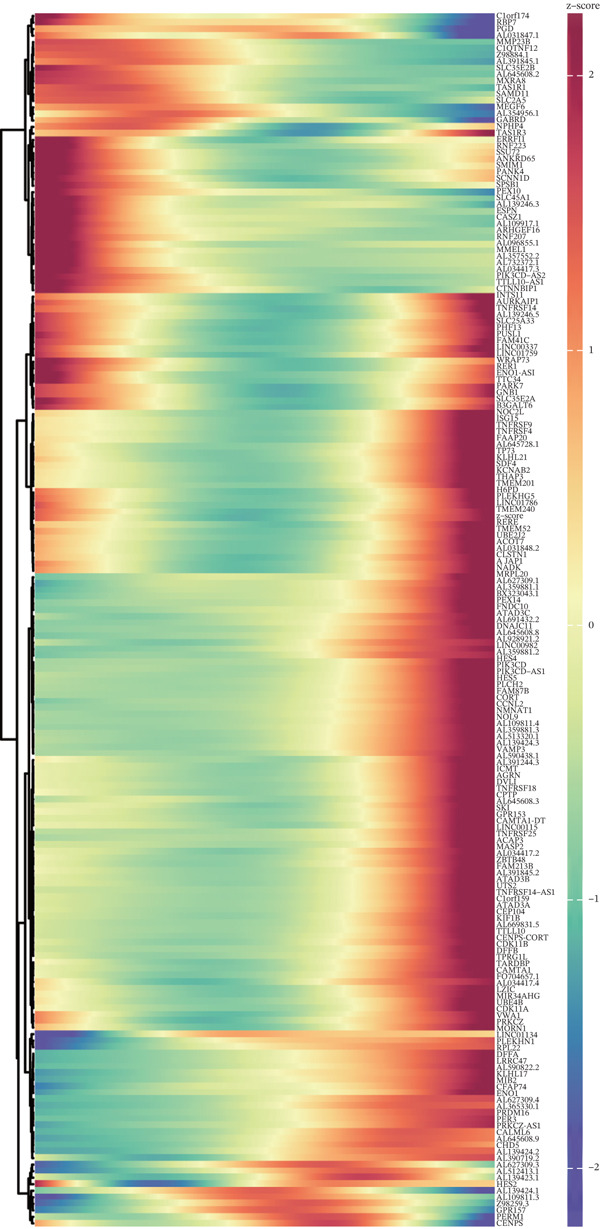
(d)

### 3.5. Knockdown and Validation of Feature Genes

DKK3 protein expression varied significantly among HET‐1A, TE‐1, and ECA109 cells (Figure 6a). DKK3 was lowest in HET‐1A, intermediate in TE‐1, and highest in ECA109 cells. Gene knockdown experiments in ECA109 cells using three siRNAs (si‐DKK3‐1, si‐DKK3‐2, and si‐DKK3‐3) showed that si‐DKK3‐1 achieved the highest knockdown efficiency and was chosen for optimal siRNA (Figure 6b). TE‐1 cells showed similar results. (Figure 6c).

Figure 6DKK3 knockdown suppresses proliferation, migration, and colony formation in ESCC cells. (a) Western blot analysis of DKK3 protein expression in HET‐1A, TE‐1, and ECA109 cells. (b) DKK3 protein levels in ECA109 cells after siRNA‐mediated knockdown with three siRNAs (si‐DKK3‐1, si‐DKK3‐2, and si‐DKK3‐3). (c) DKK3 protein levels in TE‐1 cells after knockdown with si‐DKK3‐1. (d) CCK‐8 assays comparing the proliferation of ECA109 and TE‐1 cells transfected with si‐DKK3‐1 versus negative control siRNA (si‐NC) at 0, 1, 2, and 3 days. (e) Wound‐healing assays assessing the migratory capacity of ECA109 and TE‐1 cells after DKK3 knockdown versus control. (f) Clonogenic assays showing colony formation of TE‐1 and ECA109 cells after DKK3 knockdown. Representative colony images and quantification of colony area are shown.  ^∗^p < 0.05,  ^∗∗^p < 0.01,  ^∗∗∗^p < 0.001.

**Figure:**
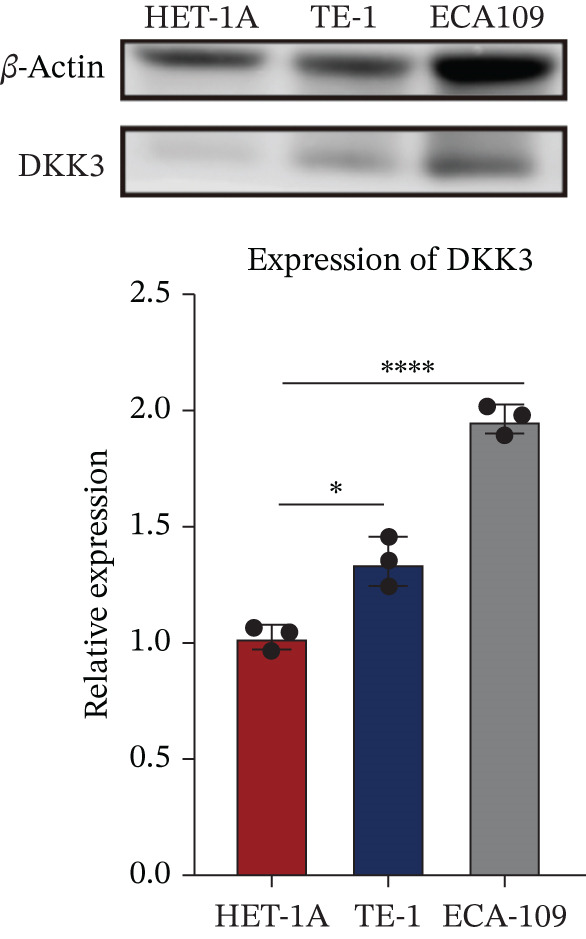
(a)

**Figure:**
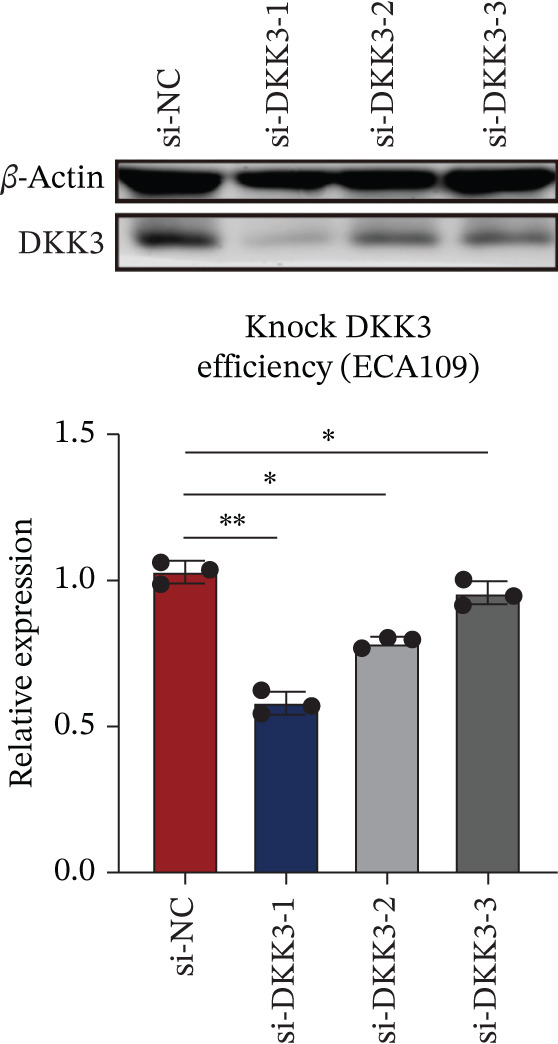
(b)

**Figure:**
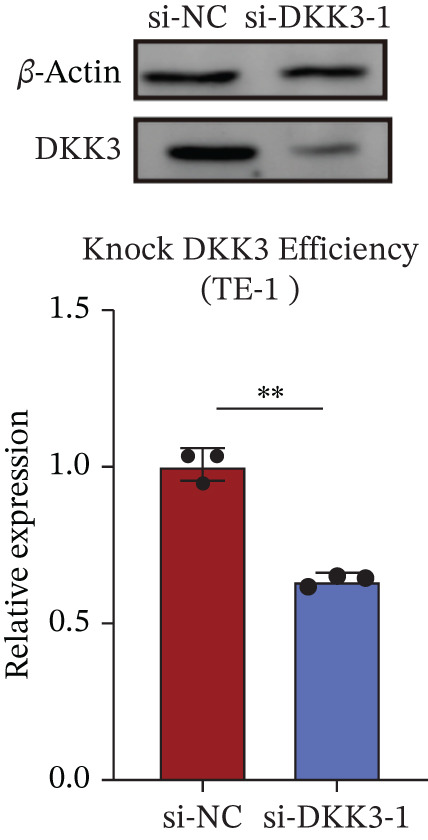
(c)

**Figure:**
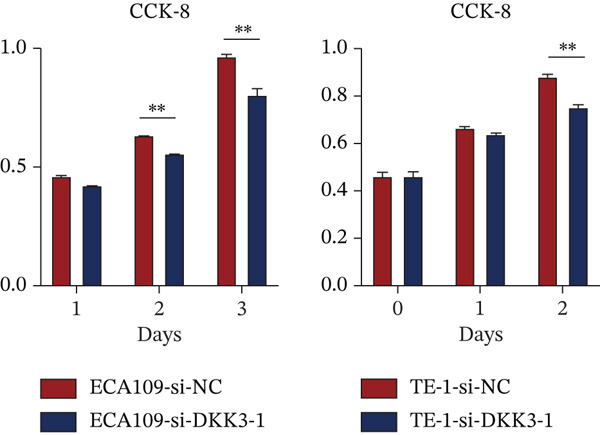
(d)

**Figure:**
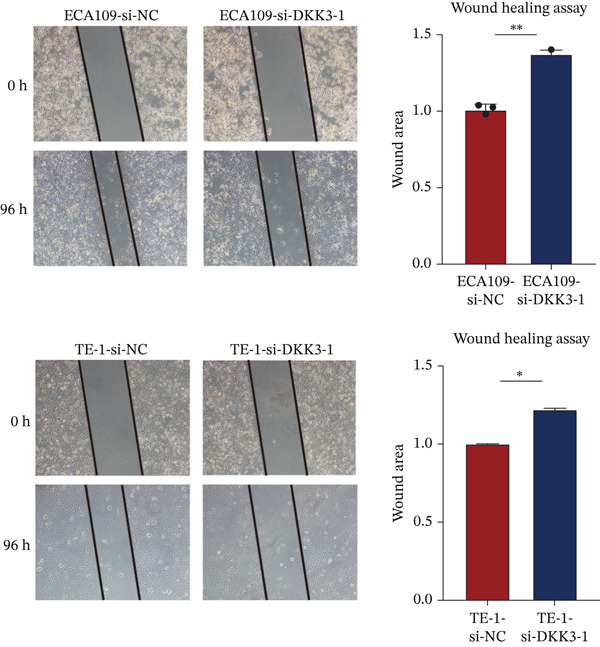
(e)

**Figure:**
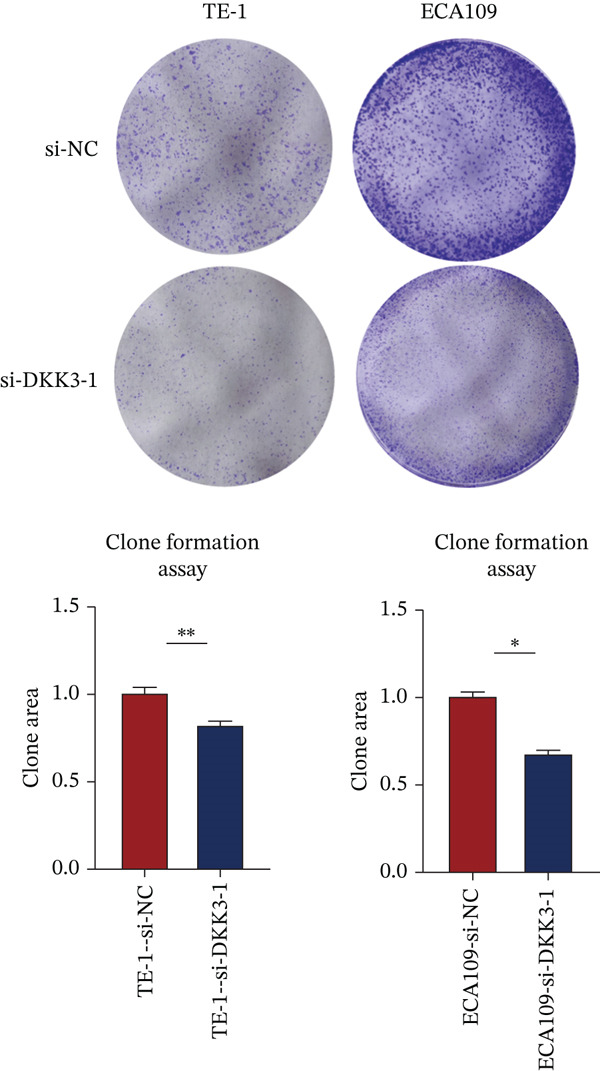
(f)

Functional experiments, including CCK‐8, wound healing, and clonogenic assays, demonstrated that DKK3 silencing markedly decreased cell growth (Figure 6d), migration (Figure 6e), and clonogenic assays (Figure 6f) in both ECA109 and TE‐1 cells. These findings highlight DKK3′s critical role in cell proliferation. Migration and clonogenic assays showed similar results, which is a potential therapeutic target for ESCC.

### 3.6. Role of Feature Genes in Animal Models

After demonstrating the role of DKK3 in cells, we further investigated its effects on tumor growth in a mouse model. We used ECA109 cells with si‐NC knockdown as the control group and ECA109 cells with si‐DKK3‐1 knockdown as the experimental group. Bioluminescence imaging showed that at 7 and 30 days, mice with DKK3 knockdown (si‐DKK3‐1 group) exhibited significantly lower luminescence intensity compared with the control group (Si‐NC group), indicating suppressed tumor cell activity (Figure 7a). The tumor volume change curve confirmed that DKK3 knockdown significantly inhibited tumor volume growth, with the black curve representing the control group and the red curve representing the DKK3 knockdown group, which showed a slower growth trend (Figure 7b). Tumor tissue images intuitively demonstrated that tumors in the si‐DKK3‐1 group were significantly smaller than those in the Si‐NC group, providing direct evidence of the inhibitory effect of DKK3 knockdown on tumor growth (Figure 7c). The tumor weight bar chart quantitatively compared the tumor weights between the two groups of mice, with results showing that the tumor weight in the si‐DKK3‐1 group was significantly lower than that in the Si‐NC group (*p* < 0.01), further confirming the effectiveness of DKK3 knockdown in inhibiting tumor growth (Figure 7d). We concluded that DKK3 knockdown effectively inhibited tumor growth in a mouse model, suggesting that DKK3 may be a potential target for anticancer therapy.

Figure 7In vivo effects of DKK3 knockdown on ESCC tumor growth. (a) In vivo bioluminescence imaging of nude mice injected with ECA109 cells transfected with si‐NC or si‐DKK3‐1 at Days 7 and 30. The color scale indicates luminescence intensity, reflecting tumor cell activity. Mice in the si‐DKK3‐1 group show markedly reduced luminescence compared with the si‐NC group, indicating suppressed tumor growth. (b) Tumor growth curves of xenografts derived from ECA109 cells transfected with si‐NC (black) or si‐DKK3‐1 (red). DKK3 knockdown significantly reduced tumor volume over time. (c) Representative photographs of excised tumors from si‐NC and si‐DKK3‐1 groups at the experimental endpoint. (d) Tumor weight comparison between the two groups. Bar plots show mean tumor weights with statistical significance indicated.  ^∗^p < 0.05,  ^∗∗^p < 0.01,  ^∗∗∗^p < 0.001.

**Figure:**
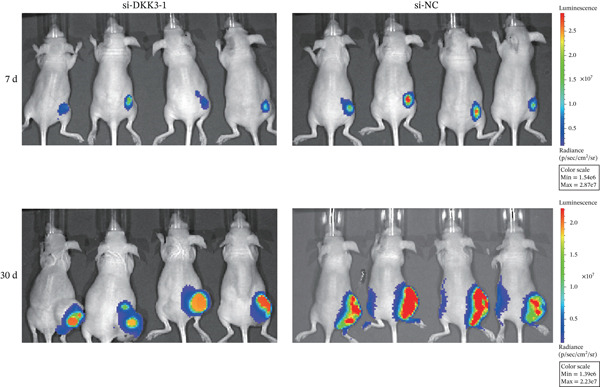
(a)

**Figure:**
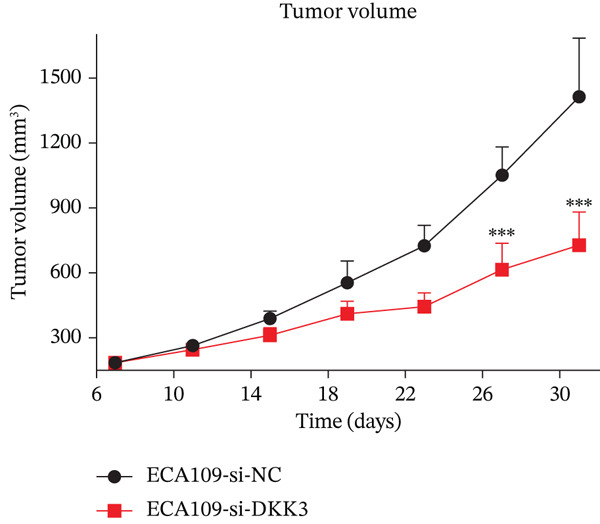
(b)

**Figure:**
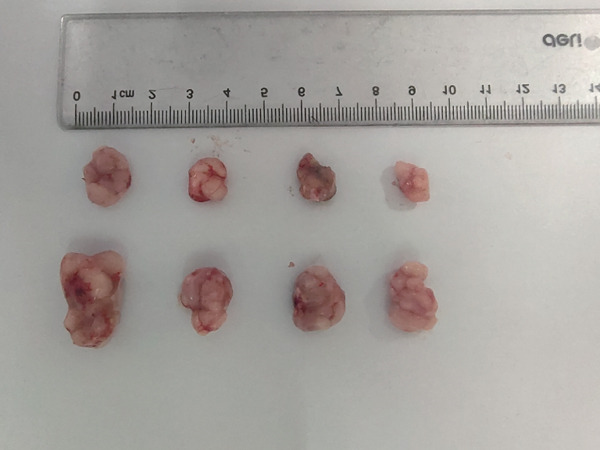
(c)

**Figure:**
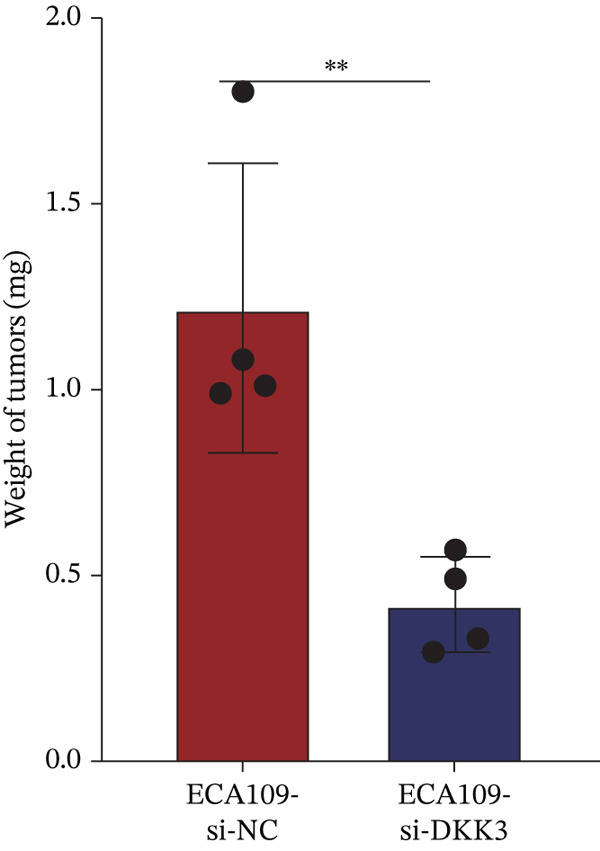
(d)

### 3.7. Analysis and Validation of Enrichment Pathways

To explore DKK3′s downstream mechanisms, we performed GO and pathway enrichment analyses on DKK3‐correlated genes in the TCGA database (Figure 8a). GO analysis covered BP, cellular components (CC), and molecular functions (MF), with bar lengths and colors indicating gene counts and adjusted *p* values, respectively. Key terms like extracellular matrix organization and collagen fiber organization showed high significance. Pathway enrichment analysis highlighted the cytoskeleton and PI3K–AKT signaling pathways.

Figure 8Integrative GO/KEGG and molecular validation analyses identify PI3K–AKT as a key DKK3‐mediated pathway in ESCC. (a) GO and KEGG enrichment analysis of DKK3‐associated DEGs. GO terms are grouped into biological process (BP), cellular component (CC) and molecular function (MF). In GO bar plots, bar length reflects gene counts and color intensity reflects *q*‐values. In KEGG dot plots, dot size represents gene counts and color indicates *q*‐value significance. (b) Epithelial cells stratified into DKK3‐high and DKK3‐low groups using an expression threshold of 0.5. (c) Pathway enrichment analysis comparing DEGs between DKK3‐high (Cluster 1) and DKK3‐low (Cluster 2) epithelial cells. Dot color reflects *q*‐values, with redder colors indicating higher statistical significance. (d) Western blot validation showing that DKK3 knockdown reduces phosphorylated AKT (p‐AKT) and phosphorylated PI3K (p‐PI3K) levels, indicating suppression of the PI3K–AKT signaling pathway.  ^∗^p < 0.05,  ^∗∗^p < 0.01,  ^∗∗∗^p < 0.001.

**Figure:**
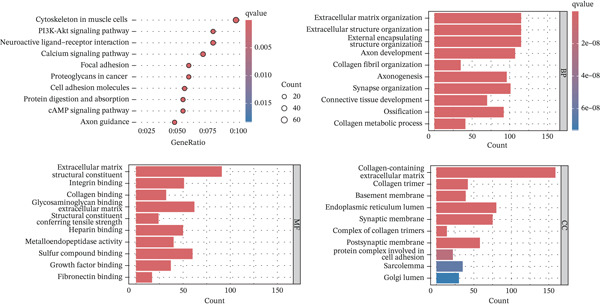
(a)

**Figure:**
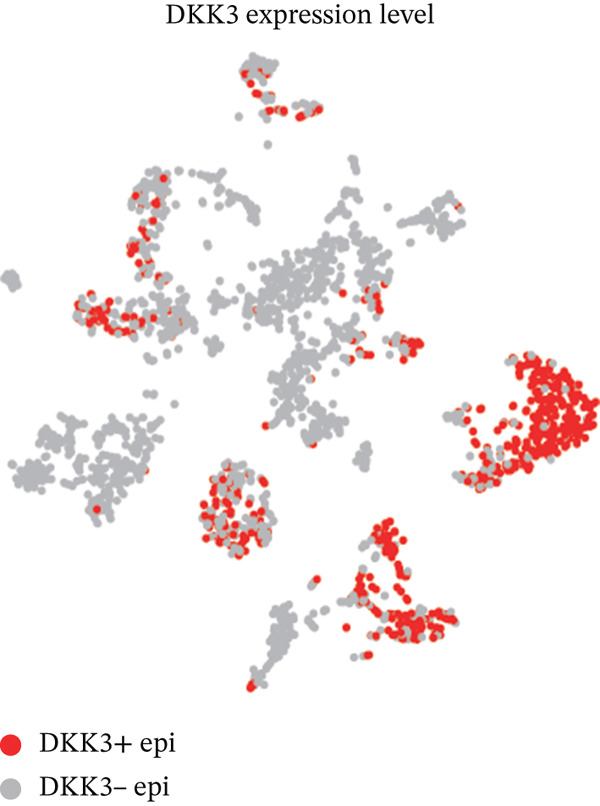
(b)

**Figure:**
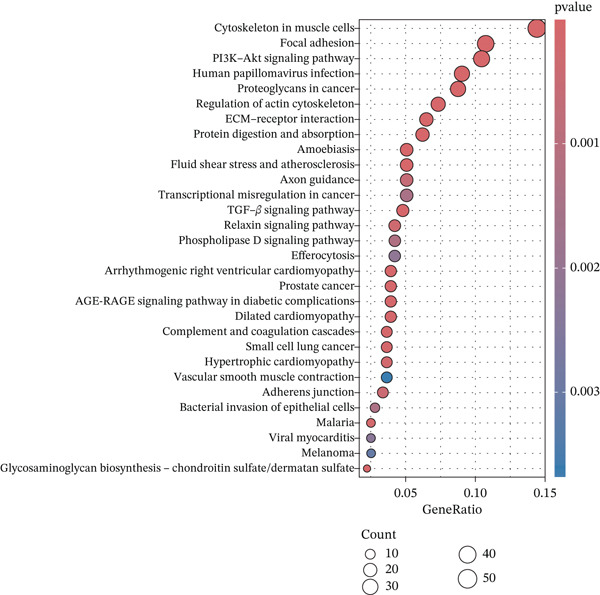
(c)

**Figure:**
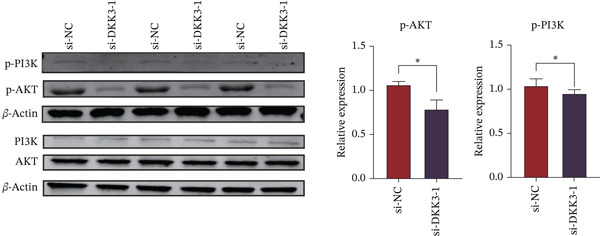
(d)

Given TCGA′s diverse cell types, we isolated epithelial cells from single‐cell data, setting a DKK3 expression threshold of 0.5 to classify high and low DKK3‐expressing cells (Figure 8b). Differential gene expression analysis and KEGG pathway enrichment showed significant phosphoinositide 3‐kinase (PI3K)–AKT pathway enrichment in high DKK3‐expressing cells (Figure 8c). Western blotting confirmed that DKK3 knockdown reduced p‐AKT and p‐PI3K levels, indicating pathway suppression (Figure 8d). These findings confirm DKK3′s role in regulating the PI3K–AKT pathway, suggesting it promotes ESCC tumor development. DKK3 knockdown inhibits this pathway, potentially curbing tumor growth.

### 3.8. MR Study Results of DKK3

In the final stage of our experiment, we performed an exhaustive evaluation to investigate the possible etiological link between the DKK3 gene and esophageal squamous cell carcinoma (ESCC). We used a forest plot to present the effect sizes and corresponding 95% confidence intervals of multiple SNPs on DKK3 gene expression (Figure 8a). In the forest plot, each SNP′s effect size is marked by a dot, and the horizontal line represents the confidence interval range. We also compared five different MR methods: IVW method, MR Egger method, weighted median method, and weighted mode method. The analysis of the combined effect sizes from these five MR methods showed high consistency, greatly enhancing the credibility of our findings (Figure 9a). A scatter plot further revealed the intrinsic relationship between the effects of SNPs on DKK3 gene expression and their effects on ESCC. In the scatter plot, different lines represent different MR methods, and the slope of the line intuitively indicates the estimated causal effect. The results strongly support the hypothesis that there is a causal relationship between DKK3 gene expression and ESCC (Figure 9b). To detect potential publication bias in the MR analysis, we used a funnel plot. In the funnel plot, each point represents the effect estimate of an SNP, and ideally, these points should be symmetrically distributed around the vertical line (representing unbiased estimation) (Figure 9c). Upon careful examination of Figure 9c, no obvious asymmetry was observed, indicating a low likelihood of publication bias in our study. Finally, we conducted a sensitivity analysis by sequentially excluding each SNP, which showed the changes in the effect sizes of the relationship between DKK3 gene expression and ESCC after the exclusion of each SNP. The results indicated that even after removing a single SNP, the findings remained unaffected, demonstrating the robustness of our study results (Figure 9d).

Figure 9Mendelian randomization analysis of the causal effect of DKK3 expression on ESCC risk. (a) Forest plot showing SNP‐specific MR estimates for the effect of genetically predicted DKK3 expression on ESCC risk, together with combined effect estimates from different MR methods (e.g., IVW, MR‐Egger, and weighted median), with 95% confidence intervals. (b) Scatter plot of SNP effects on DKK3 expression (*x*‐axis) versus ESCC risk (*y*‐axis). Regression lines represent causal effect estimates obtained from different MR methods; their slopes correspond to the inferred causal effects. (c) Funnel plot assessing the symmetry of SNP‐specific MR estimates around the pooled effect, used to evaluate heterogeneity and potential directional pleiotropy. (d) Leave‐one‐out sensitivity analysis, in which each SNP is sequentially excluded to examine its influence on the overall MR estimate for the relationship between DKK3 expression and ESCC risk.

**Figure:**
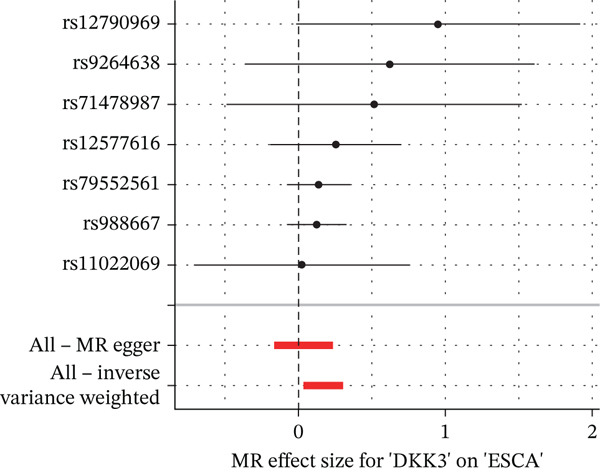
(a)

**Figure:**
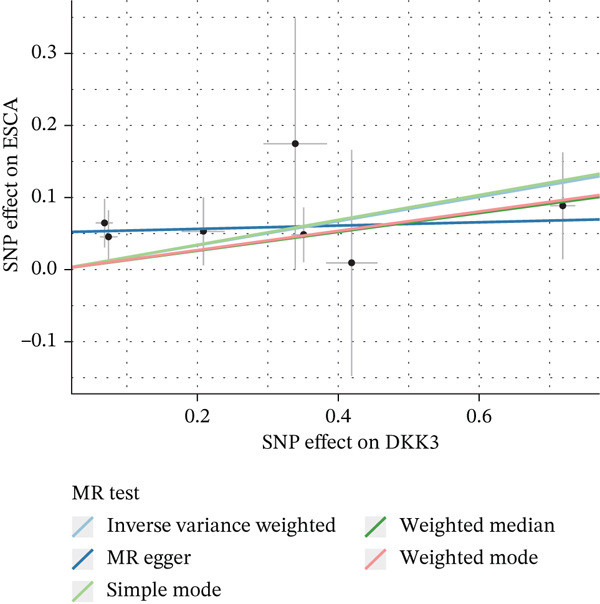
(b)

**Figure:**
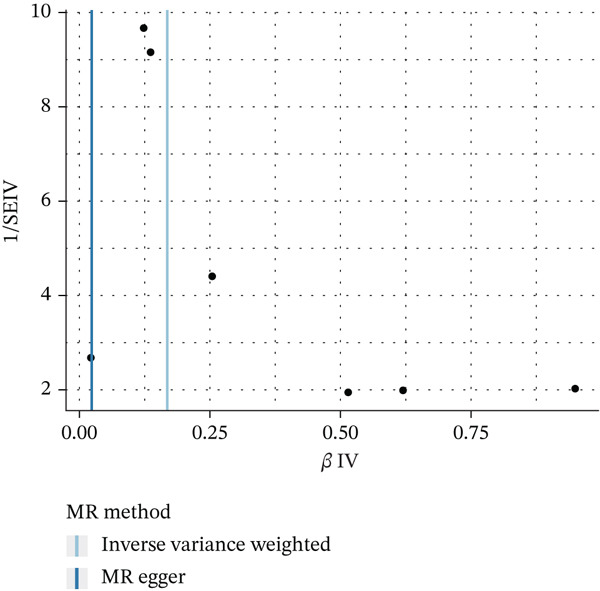
(c)

**Figure:**
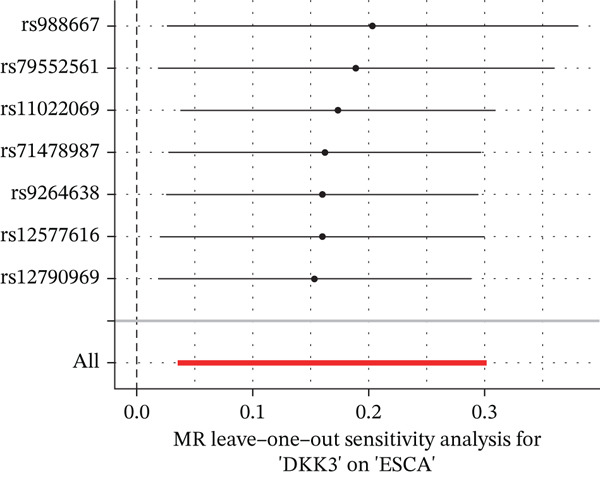
(d)

## 4. Discussion

ESCC is a malignant tumor arising from the squamous epithelium of the esophagus. In recent years, its incidence has increased, largely driven by rising rates of smoking and alcohol consumption, and it severely compromises patients′ physical health, quality of life and psychological well‐being. ESCC is closely associated with genetic susceptibility, environmental exposure, and lifestyle factors [17]. Its incidence shows marked geographic variation, with East and Southeast Asia being high‐incidence regions [18, 19]. Elucidating the pathogenesis of ESCC is therefore critical for improving treatment strategies and alleviating patient suffering.

The burden of ESCC is multifaceted. Clinically, patients frequently experience dysphagia, weight loss, and malnutrition, all of which substantially impair daily functioning and quality of life [20]. Therapeutic approaches—primarily surgical resection, radiotherapy, and chemotherapy—can further lead to postoperative complications, pain, and functional impairment [21, 22]. In parallel, the psychological status of ESCC patients is often poor, with high rates of anxiety and depression that can adversely affect treatment adherence and long‐term outcomes [23]. Thus, the impact of ESCC extends beyond physical damage to encompass profound social and psychological consequences.

From a preventive perspective, lifestyle modification remains fundamental. Smoking cessation and alcohol restriction are particularly important. MR studies have demonstrated that smoking is a major causal risk factor for ESCC, especially in individuals carrying specific genetic polymorphisms [24, 25]. However, prevention is not limited to tobacco and alcohol control; increasing the intake of fresh fruits and vegetables and reducing the consumption of very hot foods can also lower ESCC risk [26, 27].

In this study, MR supported a causal relationship between DKK3 expression and ESCC risk. By using germline variants as instrumental variables, this approach can mitigate confounding and reverse causation, providing stronger causal inference than traditional bulk association studies. Complementarily, scRNA‐seq revealed marked cellular heterogeneity in ESCC and identified biological pathways associated with DKK3 expression. At single‐cell resolution, we dissected the tumor microenvironment and found that high DKK3 expression in epithelial cells is likely linked to tumor‐promoting functions, whereas DKK3 expression in fibroblasts may influence stromal composition and function. By integrating population‐level MR with single‐cell transcriptomic and pseudotime analyses, our work provides, to our knowledge, the first example in ESCC in which a candidate gene such as DKK3 is simultaneously interrogated for genetic causality and single–cell‐level malignant evolution, extending earlier DKK3 studies that were confined to bulk expression profiling and conventional functional assays.

The rapid development of single‐cell analysis technologies has transformed tumor research by enabling investigations at the level of individual cells and uncovering intratumoral heterogeneity and microenvironmental complexity. SCA allows in‐depth analysis of the genome, transcriptome, and proteome of single cells, facilitating the identification of genetic alterations within tumor subclones and the characterization of how microenvironmental components influence tumor progression [28–30]. Compared with bulk approaches, single‐cell methods reduce the noise introduced by sample heterogeneity and provide more precise cellular phenotyping, which is crucial for understanding tumor heterogeneity, drug resistance mechanisms, and immune evasion. ScRNA‐seq has been widely applied in oncology to delineate the transcriptomic features of diverse cell types, enabling the identification of rare populations and cancer stem‐like cells [31, 32]. Advances in microfluidic technologies have further enabled high‐throughput single‐cell isolation and profiling, offering powerful tools for cancer biology research [33, 34]. Using single‐cell platforms, researchers can also evaluate tumor cell responses to different drugs, thereby supporting anticancer drug screening and development [35, 36]. Moreover, the integration of machine learning and deep learning with single‐cell datasets has begun to reveal complex cellular behaviors and drug response patterns, opening new avenues for precision cancer therapy [37–39].

We functionally validated the role of DKK3 in ESCC through in vitro and in vivo experiments. DKK3 knockdown reduced cell viability in CCK‐8 assays, inhibited cell migration in wound‐healing assays, and decreased clonogenic capacity, indicating that DKK3 is important for ESCC cell proliferation and survival. In a nude mouse xenograft model, DKK3 depletion significantly suppressed tumor growth, further supporting its potential as a therapeutic target.

Mechanistically, functional enrichment analyses highlighted the PI3K–AKT pathway as a key mediator of DKK3‐driven effects. This pathway is a central oncogenic signaling axis that regulates tumor cell proliferation, survival, and migration [39–40]. In ESCC, alterations in the PI3K/AKT/mTOR cascade are strongly associated with patient outcomes and prognostic indicators [41], whereas in breast cancer, activation of the PI3K–AKT pathway has been tightly linked to tumor initiation and progression [42]. Consequently, inhibitors targeting PI3K–AKT signaling have shown considerable promise in multiple malignancies. In acute and chronic leukemias, PI3K/AKT/mTOR inhibitors have entered clinical studies and demonstrated the potential to improve treatment responses [43]. Combination regimens involving PI3K/AKT/mTOR inhibitors and other therapeutic modalities can further enhance response rates and clinical benefit [44–46].

The PI3K family comprises a critical group of lipid kinases classified into three main classes. Class I PI3Ks, which consist of a catalytic subunit (p110) and a regulatory subunit (p85), are the most extensively studied. The p110 subunit confers catalytic activity, whereas p85 modulates PI3K stability, localization, and activation [47]. PI3K catalyzes the conversion of phosphatidylinositol 4,5‐bisphosphate (PIP2) into phosphatidylinositol 3,4,5‐trisphosphate (PIP3), a pivotal step that enables AKT recruitment and activation at the plasma membrane. Activated AKT then orchestrates diverse downstream pathways controlling cell growth, survival, metabolism, and motility. Aberrant or excessive AKT activation often leads to uncontrolled proliferation, enhanced survival, and treatment resistance [44–48]. Accordingly, pharmacologic targeting of the PI3K–AKT axis—particularly via small‐molecule inhibitors—is emerging as a major focus of anticancer drug development [49–63].

This study has several limitations. First, MR relies on the validity of genetic instruments, which may be influenced by linkage disequilibrium and horizontal pleiotropy. Second, although scRNA‐seq provides powerful resolution of cellular heterogeneity, it cannot fully capture temporal dynamics or spatial organization of cell states. Third, in vitro and animal models help clarify phenotypic consequences of modulating DKK3 but cannot fully recapitulate the complexity of human ESCC, including immune contexture and environmental exposures. Thus, further work from multiple complementary perspectives will be required to fully elucidate the mechanisms by which DKK3 and related pathways drive ESCC.

Taken together, our genetic and functional data support DKK3 as a promising biomarker and therapeutic target in ESCC. Genetically elevated DKK3 expression was associated with increased ESCC risk, and DKK3 silencing inhibited ESCC cell growth in vitro and tumor progression in vivo, suggesting that pharmacological inhibition of DKK3 or its downstream PI3K–AKT signaling may have clinical utility. Although DKK3 functions as a tumor suppressor in some cancer types, it appears to be protumorigenic in others, including ESCC, likely reflecting context‐dependent differences in signaling networks, receptor and cofactor expression, and epigenetic regulation. Future studies incorporating PI3K inhibitors will be valuable for further testing the causal role of the DKK3/PI3K/AKT axis in ESCC. In addition, because our scRNA‐seq data lack spatial information, we cannot determine whether DKK3‐high epithelial cells cluster within particular tumor subregions, and the limited quantity and quality of xenograft tumor tissue precluded direct assessment of PI3K–AKT pathway activation in vivo. These limitations should be addressed in future work, for example by integrating spatial transcriptomics, multiplex immunohistochemistry or phospho‐proteomic profiling to more precisely map DKK3‐associated signaling in the ESCC microenvironment.

## 5. Conclusion

The findings of this study lead to the conclusion that DKK3 plays a pivotal role in the progression of ESCC, with its high expression being closely associated with the malignancy of the tumor. Through scRNA‐seq, MR analysis combined with GWAS and TCGA data identified potential genes associated with ESCC. In vitro experiments and nude mouse models confirmed the significant effects of DKK3 knockdown in inhibiting the proliferation, migration, and tumor growth of ESCC cells. KEGG pathway analysis specifically highlighted the association of DKK3 with the PI3K–AKT signaling pathway, which was subsequently validated at the cellular level.

**Nomenclature**
AKT: protein kinase B
BCA: bicinchoninic acid
CAFs: cancer‐associated fibroblasts
CCK‐8: Cell Counting Kit‐8
ddH₂O: double‐distilled water
DEGs: differentially expressed genes
DKK3: Dickkopf WNT Signaling Pathway Inhibitor 3
ESCC: esophageal squamous cell carcinoma
ECA109: human esophageal cancer cells
eQTL: expression quantitative trait loci
EMT: epithelial–mesenchymal transition
GEO: Gene Expression Omnibus
GO: Gene Ontology
GWAS: genome‐wide association study
HET‐1A: human esophageal epithelial cells
IEU OPEN GWAS: IEU open genome‐wide association study
IC50: half‐maximal inhibitory concentration
IVW: inverse variance weighted
LUAD: lung adenocarcinoma
KEGG: Kyoto Encyclopedia of Genes and Genomes
mTOR: mechanistic target of rapamycin
MR: Mendelian randomization
PBS: phosphate‐buffered saline
PI3K: phosphoinositide 3‐kinase
PMSF: phenylmethanesulfonyl fluoride
PVDF: polyvinylidene fluoride
scRNA‐seq: single‐cell RNA sequencing
SDS‐PAGE: sodium dodecyl sulfate–polyacrylamide gel electrophoresis
SNP: single nucleotide polymorphism
SPF: specific pathogen‐free
TE‐1: TE‐1 esophageal cancer cell line
T‐SNE: t‐distributed stochastic neighbor embedding
PCA: principal component analysis
PVDF: polyvinylidene Fluoride
RIPA: radio‐immunoprecipitation assay buffer
TBST: tris buffered saline with tween
TCGA: The Cancer Genome Atlas
UMAP: Uniform Manifold Approximation and Projection

## Author Contributions

Zhanghao Huang: data curation, investigation, software, methodology, project administration, resources, validation, writing—original draft, writing—review and editing. Tiegang Cao: software, methodology, and writing—original draft. You Lang Zhou: resources, and writing—original draft. Jiahai Shi: conceptualization, supervision, visualization, funding acquisition, writing—original draft. Zhanghao Huang and Tiegang Cao contributed equally to this work.

## Funding

This study was supported by Jiangsu Provincial Research Hospital (YJXYY202204); Innovation Team Project of Affiliated Hospital of Nantong University (XNBHCX31773).

## Ethics Statement

The authors are accountable for all aspects of the work in ensuring that questions related to the accuracy or integrity of any part of the work are appropriately investigated and resolved. The study was conducted in accordance with the Declaration of Helsinki (as revised in 2013). The study was approved by the Ethics Committee of the Affiliated Hospital of Nantong University (No. 2022‐L165) and informed consent was taken from all the patients.

## Consent

The authors have nothing to report.

## Conflicts of Interest

The authors declare no conflicts of interest.

## Supporting information


**Supporting Information** Additional supporting information can be found online in the Supporting Information section. Figure S1:Quality control and dimensionality reduction for single‐cell RNA‐seq data. (a) Scatter plot of mitochondrial RNA percentage (percent.mt, *y*‐axis) versus total RNA counts per cell (nCount_RNA, *x*‐axis), colored by sample identity. (b) Scatter plot of feature RNA percentage versus total RNA counts per cell (nCount_RNA), colored by sample identity. (c) Plot of highly variable genes across samples. The *x*‐axis shows the geometric mean expression level, and the *y*‐axis shows residual variance. Genes in the upper‐right region are typically the most variable. (d) Violin or box plots summarizing key QC metrics: nFeature_RNA (number of detected genes per cell), nCount_RNA (total RNA counts per cell), percent.mt (fraction of mitochondrial RNA; high values may indicate low‐quality cells) and percent. HB (fraction of hemoglobin‐related genes, used to assess potential erythrocyte contamination and data stability). (e) Principal component analysis (PCA) of cells from different patients. The *x*‐ and *y*‐axes represent the first and fortieth principal components, respectively, with colors indicating patient identity. (f) Scree plot showing the standard deviation of each principal component, illustrating the proportion of variance explained. The first few principal components typically account for most of the variation and are used for downstream analyses.

## Data Availability

The data that support the findings of this study are available on request from the corresponding author. The data are not publicly available due to privacy or ethical restrictions.
