# Supplementary figures and images for: Integrative Mendelian Randomization and Single‐Cell Pseudotime Analysis Reveal DKK3 as a PI3K–AKT‐Modulated Driver of Esophageal Squamous Cell Carcinoma
Source: Hum Mutat. 2026 Mar 24;2026:6777692. doi: 10.1155/humu/6777692 (PMC13383006; doi:10.1155/humu/6777692)

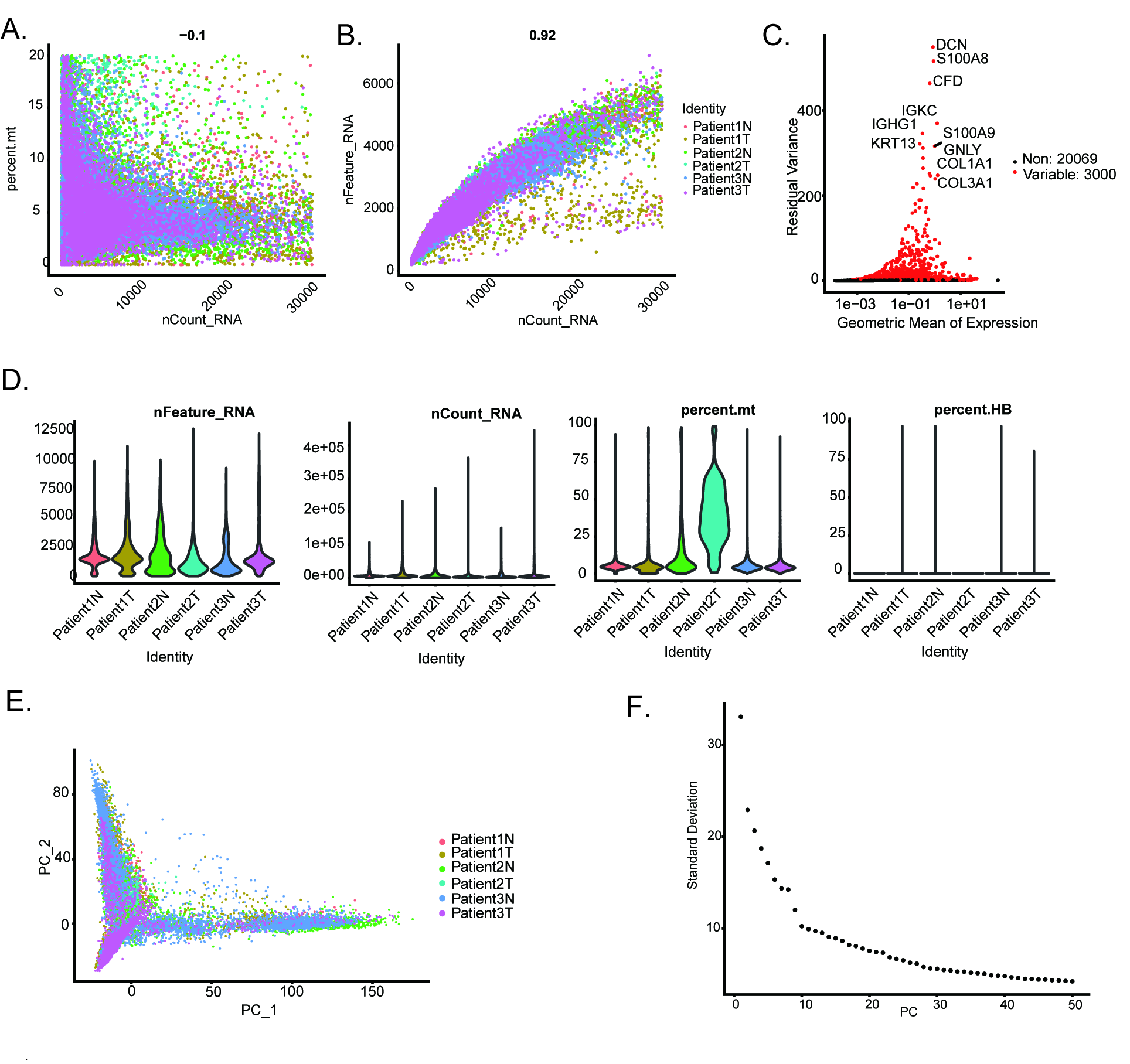

Supplement: Supplementary file 1 — Supporting Information Additional supporting information can be found online in the Supporting Information section. Figure S1:Quality control and dimensionality reduction for single‐cell RNA‐seq data. (a) Scatter plot of mitochondrial RNA percentage (percent.mt, y‐axis) versus total RNA counts per cell (nCount_RNA, x‐axis), colored by sample identity. (b) Scatter plot of feature RNA percentage versus total RNA counts per cell (nCount_RNA), colored by sample identity. (c) Plot of highly variable genes across samples. The x‐axis shows the geometric mean expression level, and the y‐axis shows residual variance. Genes in the upper‐right region are typically the most variable. (d) Violin or box plots summarizing key QC metrics: nFeature_RNA (number of detected genes per cell), nCount_RNA (total RNA counts per cell), percent.mt (fraction of mitochondrial RNA; high values may indicate low‐quality cells) and percent. HB (fraction of hemoglobin‐related genes, used to assess potential erythrocyte contamination and data stability). (e) Principal component analysis (PCA) of cells from different patients. The x‐ and y‐axes represent the first and fortieth principal components, respectively, with colors indicating patient identity. (f) Scree plot showing the standard deviation of each principal component, illustrating the proportion of variance explained. The first few principal components typically account for most of the variation and are used for downstream analyses. [file HUMU-2026-6777692-s001.tif]
